# Dental general anaesthetic receipt among Australians aged 15+ years, 1998–1999 to 2004–2005

**DOI:** 10.1186/1472-6831-8-10

**Published:** 2008-04-11

**Authors:** Lisa M Jamieson, Kaye F Roberts-Thomson

**Affiliations:** 1 Australian Research Centre for Population Oral Health, The University of Adelaide, South Australia 5005, Australia

## Abstract

**Background:**

Adults receive dental general anaesthetic (DGA) care when standard dental treatment is not possible. Receipt of DGA care is resource-intensive and not without risk. This study explores DGA receipt among 15+-year-old Australians by a range of risk indicators.

**Methods:**

DGA data were obtained from Australia's Hospital Morbidity Database from 1998–1999 to 2004–2005. Poisson regression modeling was used to examine DGA rates in relation to age, sex, Indigenous status, location and procedure.

**Results:**

The overall DGA rate was 472.79 per 100,000 (95% CI 471.50–474.09). Treatment of impacted teeth (63.7%) was the most common reason for DGA receipt, followed by dental caries treatment (12.4%), although marked variations were seen by age-group. After adjusting for other covariates, DGA rates among 15–19-year-olds were 13.20 (95% CI 12.65–13.78) times higher than their 85+-year-old counterparts. Females had 1.46 (95% CI 1.45–1.47) times the rate of their male counterparts, while those living in rural/remote areas had 2.70 (95% CI 2.68–2.72) times the rate of metropolitan-dwellers. DGA rates for non-Indigenous persons were 4.88 (95% CI 4.73–5.03) times those of Indigenous persons. The DGA rate for 1+ extractions was 461.9 per 100,000 (95% CI 460.6–463.2), compared with a rate of 23.6 per 100,000 (95% CI 23.3–23.9) for 1+ restorations.

**Conclusion:**

Nearly two-thirds of DGAs were for treatment of impacted teeth. Persons aged 15–19 years were disproportionately represented among those receiving DGA care, along with females, rural/remote-dwellers and those identifying as non-Indigenous. More research is required to better understand the public health implications of DGA care among 15+-year-olds, and how the demand for receipt of such care might be reduced.

## Background

Most of the literature pertaining to hospital-based dental care receipt under a general anaesthetic concerns children [1-3]. However, a substantial proportion of the 15+-year-old population also require such care [4-10]. This population includes those who are physically, mentally or medically compromised; those with behavioral problems such as autism or learning disabilities; those with phobias, or those who require treatment not possible under local anaesthesia settings, for example, removal of impacted wisdom teeth.

Hospital-based dental general anaesthetic (DGA) services are resource-intensive and not without risk [11]. Hospitals providing DGA services are usually in large urban centers, meaning rural/remote-dwellers may face financial pressure incurred by time off from work, childcare, travel and accommodation in order to receive such treatment. Considerable care is required in order to be fit for a DGA, including fasting before the procedure, navigating oneself through the hospital system and establishing follow-up care (driving immediately after DGA treatment is not permitted). These may be additional barriers to those already fearful or who find hospital settings stressful.

In Australia, a country with a 15+-year-old population of over 15.5 million in 2002 [12], little is known about the prevalence of adult DGA care in a hospital setting. Defining the extent of the problem is a matter of some importance, both to inform the development of appropriate DGA service provision for those aged 15+ years requiring such care, and to reorient primary dental services so that the demand for such treatment under a hospital-based general anaesthetic might be reduced.

We retrospectively examined hospital separation data collected at a national level in Australia from 1998–1999 to 2004–2005 with the purpose of exploring receipt of DGA care among 15+-year-olds in relation to age, sex, Indigenous status, location and treatment type. We aimed to test three hypotheses in this simple descriptive study: (i) DGA rates among the 15–19-year-old age-group would be higher than their older counterparts; (ii) DGA rates would be higher among those living in rural/remote locations and; (iii) there would be higher rates of extractions as opposed to more conservative procedures.

## Methods

Data on dental procedures received by those aged 15+ years admitted to public and private hospitals across all Australian states and territories were accessed from the Australian Institute of Health and Welfare National Hospital Morbidity Database from 1 July 1998 until 30 June 2005. Data were collected for administrative purposes by hospital-employed dentists and recorded in standardised ICD-10-AM (International Statistical Classification of Diseases and Related Health Problems, 10th Revision, Australian Modification) codes, which are patient record codes used throughout Australian hospitals. Because all data were de-identified and collected primarily for administrative purposes, the Human Research Ethics Committee of the University of Adelaide did not consider ethical approval to be necessary for the secondary analysis of such data. The ICD-10-AM dental procedure codes pertaining to extractions or restorations were included. Demographic information was collected and included patients' age, sex, Indigenous status and residential location. Age was broken down into 5-year groupings, up to the age of 85+ years (15 groups in total). Indigenous status was defined by a person identifying as being Aboriginal, Torres Strait Islander or both, ie the ethnicity of the first Australian inhabitants, and was indicated by self-report in a routine question about ethnic identity upon admission. Separations with Indigenous status 'not stated' were excluded from the analyses.

Residential location was measured using the Rural, Remote and Metropolitan Areas (RRMA) classification, which is an index based on Statistical Local Areas (SLA) that allocates each SLA in Australia to a category based primarily on population numbers and an index of remoteness. 'Metropolitan' is defined as any capital city or other metropolitan area with a population of > 100,000, 'rural' zones are those with a population ranging from 10–99,000 and 'remote' areas those with a population of < 10,000. For the purposes of this study, 'rural' and 'remote' zones were combined.

Estimated resident population (ERP) counts of all demographic stratifications (sex, age, Indigenous status, location) for the years 1998–1999 to 2004–2005 were provided by the Australian Bureau of Statistics. DGA incident rates (number of DGA separations for a specified strata/diagnosis divided by the ERP of the same specified strata, multiplied by 100,000) and incident rate ratios were computed along with 95% confidence intervals (95% CI) using a generalised Poisson regression model [13]. Results from the Poisson regression models are presented as non-adjusted and adjusted incident rate ratios to estimate the independent effect of each covariate on the DGA rate. Data were analysed using Intercooled Stata 8.0.

## Results

There were a total of 502,522 hospital admissions for dental care under a general anaesthetic for those aged 15+ years between 1998–1999 and 2004–2005 at a national level in Australia. The overall DGA rate was 472.79 per 100,000. Impacted teeth was the most prevalent diagnosis (63.7 percent), followed by dental caries (12.4 percent; Table 1). However, there was marked variation in treatment received by age-group, with nearly 80 percent of those aged 15–24 years receiving DGA care for treatment of impacted teeth, which steadily decreased across age-groups to less than 10 percent of those aged 85+ years. The converse was observed for the treatment of dental caries, with less than 3 percent of 15–19 year olds receiving DGA care for this reason, which steadily increased across age-groups to 46.2 percent of those aged 85+ years. Impacted teeth was the most common diagnosis for those aged 15–44 years, followed by dental caries among those aged 25–44 years. Among those aged 44+ years, dental caries was the most common principle diagnosis, followed by impacted teeth (except for those aged 80+ years, where periapical abscess was the 2^nd ^most common diagnosis). Disturbances in tooth eruption or anomalies of tooth position were the 2^nd^–4^th ^most common reason for DGA receipt among those aged 15–34 years, while periapical abscess was the 3^rd ^most common reason for DGA receipt among their 35–79-year-old counterparts. Pathological fracture of tooth was the 4^th ^most common reason for DGA receipt among those aged 45–69 years.

**Table 1:** Ten most common principle diagnoses for DGA receipt among Australians aged 15+ years; 1998–1999 to 2004–2005 by age-group (percent) (top five diagnoses per agegroup highlighted in bold)

	AGE GROUP (years)															
PRINCIPLE DIAGNOSIS	Total	15–19	20–24	25–29	30–34	35–39	40–44	45–49	50–54	55–59	60–64	65–69	70–74	75–79	80–85	85+
Impacted teeth	**63.7**	**79.3**	**79.3**	**71.6**	**62.7**	**49.8**	**37.2**	**27.8**	**21.8**	**18.1**	**15.4**	**14.4**	**8.7**	**9.9**	**9.2**	**7.1**
Dental caries, unspecified	**12.4**	**2.7**	**3.9**	**9.1**	**15.1**	**22.3**	**28.5**	**32.6**	**33.3**	**34.8**	**34.7**	**35.2**	**43.2**	**41.6**	**43.6**	**46.2**
Disturbances in tooth eruption	**3.9**	**5.5**	**4.6**	**4.2**	**3.2**	**2.7**	**2.1**	1.6	1.4	1.1	0.9	1.3	0.7	0.8	0.8	0.6
Anomalies of tooth position	**3.2**	**4.8**	**3.6**	**3.2**	**2.8**	**2.4**	**2.1**	1.3	1.1	0.8	0.7	0.6	0.4	0.4	0.4	0.2
Periapical abscess w/o sinus	**2.4**	0.3	0.9	**1.6**	**2.7**	**4.0**	**5.5**	**6.4**	**6.9**	**7.4**	**7.7**	**7.1**	**8.6**	**7.9**	**9.6**	**8.5**
Chronic periodontitis	1.2	0.7	**1.3**	1.3	1.3	1.4	1.4	1.5	1.6	1.6	1.5	1.2	0.6	0.7	0.5	0.5
Embedded teeth	1.0	**1.2**	1.2	1.3	1.1	0.9	0.6	0.5	0.4	0.4	0.3	0.3	0.3	0.3	0.1	0.3
Disorder of teeth, unspecified	1.0	0.5	0.5	0.7	0.8	1.2	1.5	**1.9**	**2.1**	**2.7**	**2.7**	**2.9**	**3.6**	**3.5**	**3.6**	**3.5**
Other disorders of teeth	0.7	0.3	0.3	0.5	0.6	0.9	1.2	1.5	1.8	1.8	2.1	2.0	2.2	2.3	1.9	2.3
Pathological fracture of tooth	0.7	0.0	0.1	0.1	0.4	0.8	1.6	**2.3**	**3.2**	**3.1**	**3.3**	**3.4**	**3.0**	**2.8**	**3.0**	**3.1**

The overall DGA rate was higher among those aged 15–19 years (1428.41 per 100,000) and lower among those aged 85+ years (117.18 per 100,000) (Table 2). After adjusting for other covariates, those aged 15–19 years had 13.20 times the DGA rate of those aged 85+ years. Females had 1.46 times the rate of males, while those living in rural/remote locations had 2.70 times the rate of their metropolitan-dwelling counterparts. Those identifying as non-Indigenous had 4.88 times the DGA rate of their Indigenous counterparts.

**Table 2:** DGA rates, unadjusted incidence rate ratios and generalised adjusted incident rate ratios among Australians aged 15+ years; 1998–1999 to 2004–2005

Variable	Dental morbidity	Rate per 100,000	95% CI*	Unadjusted rate ratio	95% CI*	Adjusted rate ratio†	95% CI*
Total	502522	472.79	471.50–474.09				
Age-group (years)							
15–19	134572	1428.41	1420.78–1436.09	12.19	11.68–12.72	13.20	12.65–13.78
20–24	123453	1317.19	1309.83–1324.58	11.24	10.77–1.73	12.36	11.85–12.90
25–29	67967	662.79	657.80–667.81	5.66	5.42–5.90	6.14	5.88–6.41
30–34	44984	435.51	431.49–439.57	3.72	3.56–3.88	3.95	3.78–4.12
35–39	30145	287.79	284.54–291.06	2.46	2.35–2.57	2.58	2.47–2.69
40–44	23375	238.98	235.93–242.07	2.04	1.95–2.13	2.13	2.04–2.22
45–49	18822	195.82	193.03–198.65	1.67	1.60–1.75	1.75	1.67–1.82
50–54	16261	182.89	179.40–185.02	1.55	1.49–1.63	1.62	1.55–1.70
55–59	12585	173.32	170.31–176.38	1.48	1.41–1.55	1.54	1.47–1.61
60–64	8467	146.20	143.12–149.35	1.25	1.19–1.31	1.29	1.23–1.35
65–69	6347	238.44	234.15–242.80	2.03	1.94–2.13	2.10	2.01–2.20
70–74	5382	228.46	224.05–232.96	1.95	1.86–2.04	2.02	1.93–2.11
74–79	4783	131.91	128.21–135.71	1.13	1.07–1.18	1.17	1.11–1.23
80–84	3201	136.38	131.72–141.21	1.16	1.10–1.23	1.20	1.13–1.26
85+ (ref)	2172	117.18	112.34–122.24				
							
Sex							
Male (ref)	208178	395.17	393.49–396.86				
Female	294344	549.38	547.41–551.36	1.39	1.38–1.40	1.46	1.45–1.47
							
Location							
Metropolitan (ref)	371312	402.54	401.26–403.82				
Rural/Remote	294344	960.97	955.77–966.21	2.39	2.37–2.40	2.70	2.68–2.72
							
Indigenous status							
Indigenous (ref)	4280	210.96	204.70–217.40				
Non-Indigenous	498242	477.77	476.45–479.09	2.26	2.20–2.33	4.88	4.73–5.03

*95% Confidence Intervals computed under standard large sample Poisson assumptions [13]

†Generalised Poisson regression model rate ratio estimates are adjusted for all other covariates

When the DGA rate was separated into distinct procedures, the rate for one or more extractions (extraction 1+) was 461.88 per 100,000, while that for one or more restorations (restoration 1+) was 23.55 per 100,000 (note that the two procedures are not mutually exclusive, that is, it is possible for an individual to have had one or more extractions as well as one or more restorations) (Table 3). After adjusting for potential confounders, those aged 15–19 years had 13.28 times the extraction 1+ rate of their 85+-year-old counterparts, and females had 1.46 times the rate of males. Those living in rural/remote locations had 2.70 times the extraction 1+ rate of those living in metropolitan settings, while those identifying as non-Indigenous had 4.90 times the rate of those identifying as Indigenous. After adjusting for other covariates, those aged 15–19 years had 9.14 times the restoration 1+ rate of those aged 85+ years. Females had 1.42 times the rate of males, while those living in rural/remote locations had 3.96 times the rate of their metropolitan-dwelling counterparts. Extraction 1+ rates among those identifying as non-Indigenous were 1.71 times higher than rates of those identifying as Indigenous.

**Table 3:** DGA rates by extraction 1+ and restoration 1+, unadjusted incidence rate ratios and generalised adjusted incident rate ratios among Australians aged 15+ years; 1998–1999 to 2004–2005

	Extractions 1+				Restorations 1+			
Variable	No.	Rate per 100,000 (95% CI)*	Unadjusted rate ratio (95% CI)*	Adjusted rate ratio† (95% CI)*	No.	Rate per 100,000 (95% CI)*	Unadjusted rate ratio (95% CI)*	Adjusted rate ratio† (95% CI)*
Total	490851	461.88 (460.60–463.17)			24911	23.55 (23.27–23.85)		
Age-group (years)								
15–19	133326	1415.22 (1407.62–1422.86)	12.26 (11.75–12.80)	13.28 (12.72–13.86)	2857	33.50 (32.35–34.69)	8.68 (6.86–10.99)	9.14 (7.23–11.57)
20–24	122491	1306.96 (1299.64–1314.33)	11.33 (10.85–1.82)	12.46 (11.93–13.00)	3038	32.40 (31.27–33.58)	8.40 (6.64–1-.63)	9.12 (7.21–11.55)
25–29	66837	651.76 (646.82–656.74)	5.65 (5.41–5.90)	6.13 (5.87–6.40)	3372	32.87 (31.78–34.00)	8.52 (6.74–10.78)	9.09 (7.19–1.50)
30–34	43694	423.10 (419.14–427.10)	3.67 (3.51–3.83)	3.89 (3.73–4.07)	3373	32.62 (31.54–33.75)	8.46 (6.69–10.70)	8.78 (6.94–11.11)
35–39	28744	274.45 (271.29–277.66)	2.38 (2.28–2.49)	2.50 (2.39–2.61)	3174	30.31 (29.27–31.39)	7.86 (6.21–9.94)	8.05 (6.36–10.18)
40–44	21933	224.22 (221.26–227.22)	1.94 (1.86–2.03)	2.03 (1.94–2.12)	2833	29.02 (27.97–30.11)	7.52 (5.94–9.52)	7.60 (6.00–9.62)
45–49	17506	182.19 (179.50–184.91)	1.58 (1.51–1.65)	1.65 (1.58–1.72)	2203	22.93 (21.99–23.91)	5.95 (4.69–7.53)	6.08 (4.80–7.70)
50–54	15152	169.83 (167.14–172.57)	1.47 (1.41–1.54)	1.54 (1.47–1.61)	1643	18.43 (17.56–19.35)	4.78 (3.77–6.06)	4.89 (3.86–6.20)
55–59	11829	162.90 (159.98–165.87)	1.41 (1.35–1.48)	1.47 (1.40–1.54)	1044	14.39 (13.54–15.29)	3.73 (2.93–4.74)	3.79 (2.98–4.82)
60–64	8057	139.12 (136.09–142.19)	1.21 (1.15–1.26)	1.24 (1.18–1.30)	507	8.79 (8.06–9.59)	2.28 (1.78–2.92)	2.29 (1.79–2.94)
65–69	6094	230.06 (225.85–234.35)	1.99 (1.90–2.09)	2.06 (1.96–2.15)	307	10.96 (10.07–11.93)	2.84 (2.22–3.64)	2.88 (2.25–3.68)
70–74	5224	224.16 (219.79–228.62)	1.94 (1.85–2.04)	2.01 (1.92–2.11)	229	7.40 (6.64–8.25)	1.92 (1.48–2.48)	1.96 (1.52–2.54)
74–79	4684	129.19 (125.53–132.95)	1.12 (1.06–1.18)	1.16 (1.10–1.22)	171	4.35 (3.72–5.09)	1.13 (0.85–1.49)	1.17 (0.88–1.55)
80–84	3142	133.93 (129.31–138.72)	1.16 (1.10–1.23)	1.19 (1.13–1.26)	89	3.87 (3.14–4.75)	1.00 (0.73–1.37)	1.03 (0.75–1.40)
85+	2138	115.39 (110.59–120.40)			71	3.86 (3.06–4.87)		
								
Sex								
Male (ref)	203422	386.25 (384.58–387.91)			10371	20.02 (19.65–20.41)		
Female	287429	536.52 (534.57–538.47)	1.39 (1.38–1.40)	1.46 (1.45–1.46)	14540	27.04 (26.60–27.48)	1.35 (1.32–1.38)	1.42 (1.38–1.45)
								
Location								
Metropolitan (ref)	362804	393.36 (392.10–394.63)			16178	17.42 (17.16–17.69)		
Rural/Remote	287429	938.05 (932.90–943.22)	2.38 (2.37–2.40)	2.70 (2.69–2.72)	8585	66.17 (64.82–67.56)	3.80 (3.70–3.90)	3.96 (3.86–4.07)
								
Indigenous status								
Indigenous (ref)	4178	206.23 (200.04–212.60)			318	30.21 (27.90–32.72)		
Non-Indigenous	486673	466.74 (465.44–468.05)	2.26 (2.20–2.33)	4.90 (4.75–5.06)	24593	23.43 (23.14–23.72)	0.76 (0.72–0.84)	1.71 (1.57–1.85)

*95% Confidence Intervals computed under standard large sample Poisson assumptions [13]

†Generalised Poisson regression model rate ratio estimates are adjusted for all other covariates

**Figure 1:**
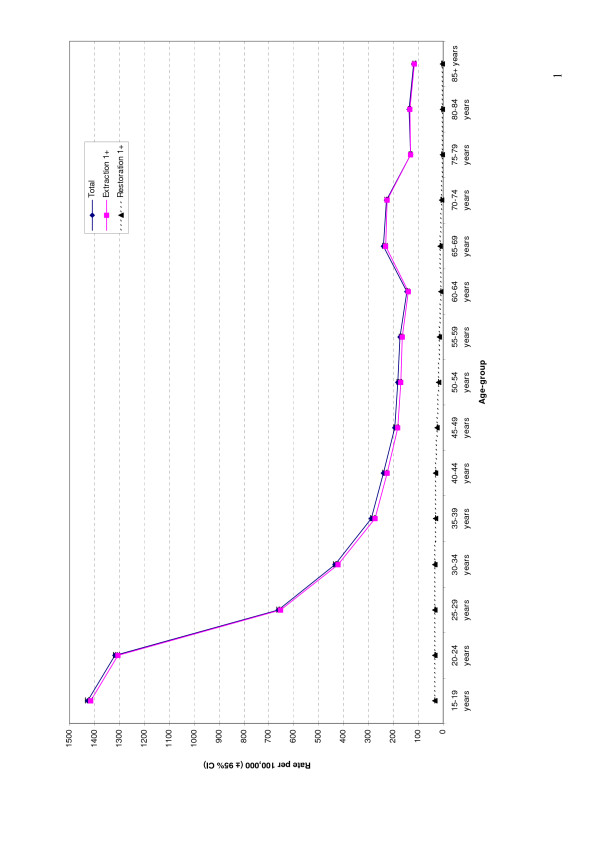
DGA rates by age-group and procedure, 1998–1999 to 2004–2005.

The overall DGA rate across age-groups closely matched the extraction 1+ rate (Figure 1). The restoration 1+ rate followed a separate, and much lower, trend. DGA rates were the highest among those aged 15–19 years, and followed a downward curvilinear trajectory until the 60–64 year age-group, when a slight increase was observed. The rate fell again among the 75–79 year age-group, and remained steadily at this level across to the 85+ year age-group.

## Discussion

This study has shown that treatment of impacted teeth was the most common reason for DGA receipt among 15+ year-old Australians between 1998–1999 and 2004–2005, followed by dental caries treatment. However, the treatment received differed markedly when considered by age. DGA rates were the highest among those aged 15–24 years, and followed a downward trajectory until the 60–64 year age-group, when a slight increase was observed.

It is important to consider the validity and coverage of the information used in this analysis. Data were collected by hospital administrators who may not have, for whatever reasons, the same rigour and thoroughness of researchers using the data for primary investigative purposes. Although data was collected from all hospital-administrated DGAs, it did not include procedures performed outside hospital settings. However, with increasing concerns of litigation and safety, non-hospital administrated DGAs in Australia are few.

That DGA rates were higher among 15–24-year-olds in our study was unsurprising given evidence in the literature that suggests that those in this age-group are more likely to have their wisdom or other impacted teeth removed than their older counterparts [9,14]. However, a sizeable proportion of the 25+-year-old population still received DGA care that, up until the age of 44 years, was predominantly for the removal of impacted teeth. From the age of 45+ years, the most common reason for DGA care was for treatment of dental caries, indicating that removal of impacted teeth among this age-group is perhaps more dangerous or that people in this age-group have already had any problematic impacted teeth removed.

It is important to consider whether the removal of wisdom teeth constitutes a public health problem and, given the scarcity of health resources, if there is an argument that a substantial proportion of such teeth need not be removed at all. Brickley and Shepherd [15] explored the rationale of lower third molar removal, based on the United State's National Institutes of Health (NIH) criteria, and concluded that while the most frequent reason for third molar removal in their study was pericoronitis, the next most frequent reason was that the contralateral tooth had been scheduled for removal under general anaesthesia. Of all the teeth scheduled for removal, only 59 percent were justified according to NIH criteria. Some 34 percent of patients might have avoided surgery altogether if NIH criteria had been the basis for intervention and a further 30 percent could have been scheduled for removal of one tooth only. These findings suggest that criteria for removal other than NIH criteria were being applied, resulting in unnecessary treatment under a general anaesthetic. Given that conservative care in a dental clinic setting is almost always preferable to treatment under a hospital-based general anaesthetic, it may be important for policy makers to consider the reasons for DGA care among the 15+-year-old population and to implement changes that encourage less costly and more conservative service provision measures for teeth that may not necessarily require removal.

There is no evidence to suggest why female DGA rates were one and a half times those of their male counterparts, although our findings are supported by the literature [16,17]. Possible explanations include females being more motivated to receive such care or being more fearful about treatment for potential traumatic procedures under local anaesthetic settings. Females, in general, receive dental care more frequently than their male counterparts [18], meaning our findings may be an extension of this general trend.

It is interesting to note that while rural/remote dwellers had 2.7 times the DGA rate of their metropolitan-dwelling counterparts overall, and when one or more extractions were considered, this rate increased to 3.8 when restorations only were considered. This may reflect disparities in access to dental services, with dental personnel for adult populations in rural/remote areas in Australia being scarce [19,20]. Vast geographic distances between rural townships in Australia and limited dental personnel in remote communities mean rural-dwellers may put off dental care until it can no longer be ignored; meaning teeth with carious lesions that may have been treated conservatively in the early stages require more radical treatment once the infection has spread. Rural-dwelling persons in Australia are, on the whole, more socio-economically deprived than metropolitan-dwelling persons [21], with the association between low socio-economic status and dental disease experience being well established [22]. Oral health expectations of rural-remote dwellers may also differ from their metropolitan-dwelling counterparts, with requests for teeth to be retained or removed influenced by access to dental services, a person's compliance with oral hygiene, oral health experience of other family members, priority of oral health to family members and familial dental health awareness [23].

It was unsurprising that non-Indigenous DGA rates were higher than their Indigenous counterparts, given that the anatomical features of many Indigenous Australians means that wisdom or other teeth are less likely to be impacted [24-26]. However, it is important to consider that Indigenous adults are generally reluctant to present for dental care, despite having high levels of untreated dental disease [27]. There is also a general reluctance of Indigenous Australians to present for hospital-based care [28,29], meaning our findings may be an under-estimation of the true need for this type of service provision – particularly for treatment of dental pathology.

There is limited evidence of the demand for adult DGA service provision in Australia. However, a national-level investigation of the Canadian adult population demonstrated a substantial need for such care, with 12 percent of those surveyed being 'definitely interested' in GA for their dentistry and a further 42 percent being 'interested depending on cost' [6]. This increased to 31 percent being interested, and 54 percent being interested depending on cost, when only those with high fear were considered. Dental fear may be one reason why DGA rates persisted through the age-groups examined in our study. Armfield and colleagues [30] reported that the prevalence of high dental fear at a national level in Australia was 16 percent, with this proportion increasing when females only were considered. Those aged 40–64 years had the highest prevalence of high dental fear, while people from higher socio-economic status groups generally had less fear. Although not examined, it is interesting to speculate if perhaps high fear patients were also more likely to desire dental care under hospital-based circumstances. More surveys on the demand and reasons for DGA care among adult populations would be useful.

Our demographic data reflects that reported from an investigation of practice patterns of oral and maxillofacial surgeons (those most qualified to remove impacted teeth) in Australia [31], where the largest proportion of clients seen (29 percent) were in the 18–24-year age group, and a slightly higher proportion (56 percent) were female. The main procedures conducted were for surgical removal of unerupted or partly erupted teeth, thus reflecting our study findings. The predominant location of services provided in both the public and private setting was a hospital theatre. While the main procedure among those aged 18–64 years was for dentoalveolar purposes (ie impaction), among those aged 65+ years, pathology was the predominant reason for a DGA. These findings are directly comparable with our study, with those aged 65+ years receiving DGA care, and most notably, extractions, because of underlying pathology (for example, dental caries) as opposed to treatment for impaction.

## Conclusion

Our study has shown that receipt of DGA care among the 15+-year-old Australian population is largely for treatment of impacted teeth, and that differences exist in relation to age, sex, Indigenous status, location and type of care received. Reasons for our findings are likely to be complex but may include access to care issues, limited resources, high treatment needs and behavioural factors. More research is required to better understand the public health implications of DGA receipt among 15+-year-olds, and to determine how demand for the need of such care might be reduced. The findings have public health and dental service provision relevance both in Australia and other nations.

## Competing interests

The author(s) declare that they have no competing interests.

## Authors' contributions

LMJ obtained and analysed the data from the Australian Institute of Health and Welfare Hospital Morbidity Database, and drafted the manuscript. KRT participated in the design of the study and completion of the manuscript. All authors read and approved the final manuscript. There were no sources of funding for this study.

## Pre-publication history

The pre-publication history for this paper can be accessed here:


